# Toll-like Receptors in Ovarian Cancer as Targets for Immunotherapies

**DOI:** 10.3389/fimmu.2014.00341

**Published:** 2014-07-22

**Authors:** Maria Muccioli, Fabian Benencia

**Affiliations:** ^1^Molecular and Cell Biology Program, Ohio University, Athens, OH, USA; ^2^Department of Biomedical Sciences, Heritage College of Osteopathic Medicine, Ohio University, Athens, OH, USA

**Keywords:** ovarian cancer, toll-like receptors, tumor microenvironment, immunotherapy, pattern-recognition receptors

## Abstract

In the last decade, it has become apparent that toll-like receptor (TLR) signaling can play an important role in ovarian cancer (OC) progression. Interestingly, TLR activation in immune cells can help activate an anti-tumor response, while TLR signaling in tumor cells themselves is often associated with cancer-promoting inflammation. For example, it has been shown that TLR activation in dendritic cells can result in more effective antigen presentation to T cells, thereby favoring tumor eradication. However, aberrant TLR expression in OC cells is associated with more aggressive disease (likely due to recruitment of pro-tumoral leukocytes to the tumor site) and has also been implicated in resistance to mainstream chemotherapy. The delicate balance of TLR activation in the tumor microenvironment in different cell types altogether help shape the inflammatory profile and outcome of tumor growth or regression. With further studies, specific activation or repression of TLRs may be harnessed to offer novel immunotherapies or adjuvants to traditional chemotherapy for some OC patients. Herewith, we review recent literature on basic and translational research concerning therapeutic targeting of TLR pathways for the treatment of OC.

## Introduction

Ovarian cancer (OC) has the most devastating death rate of gynecological cancers with only 44% of women surviving 5 years after diagnosis (1–4). The low long-term survival statistics are in part due to lack of efficient screening technology; by the time symptoms occur, most patients exhibit advanced-stage disease (over 60% of OC is diagnosed after distant metastasis). The survival rates decrease with each later stage of diagnosis with only a 27% 5-year relative survival rate for distantly metastasized tumors, highlighting the need for more efficacious treatments for advanced OC (4). Today, the standard of therapy includes surgery (hysterectomy and bilateral salpingooophorectomy) and several rounds of platinum- or taxane-based chemotherapy (1–3). Chemotherapy typically produces significant side effects, such as nausea, weight loss, fatigue, and alopecia, largely a result of toxicity of the treatment to healthy cells (5). Moreover, many cancers become resistant to treatment, further warranting the development of additional and more tumor-specific therapies. Thus, although chemotherapy remains the gold-standard of OC management, replacement as well as adjuvant treatments are in process of intensive investigation. As it is well-established that the immune system (if properly functioning) can fight tumor growth, tumor immunology research and immunotherapy clinical trials are taking center-stage in the quest for better clinical outcomes for late-staged OC (6–9).

As aggressive OC often correlates with an immunosuppressive leukocyte population in the tumor environment, efforts to modulate these cells to potentiate an anti-tumor immune response are ongoing (10–12). Of particular interest to the processes of tumor-infiltration by immune cells and their activation are the toll-like receptors (TLRs), pattern-recognition receptors (PRRs) that ligate conserved pathogen-associated molecular patterns (PAMPs) such as bacterial lipopolysaccharide (LPS) or viral dsRNA (13–20). TLR expression is well-established in immune cells, such as macrophages and dendritic cells (DCs), where upon PAMP recognition, an inflammatory response occurs, activating numerous transcription factors, such as NF-kB and IRF 3/7 (21–23). Cytokine and chemokine secretion subsequently ensues, further activating inflammation and stimulating the adaptive immune response. In fact, TLR activation in leukocytes (e.g., DCs) can trigger a shift in the inflammatory profile of the tumor site by decreasing immunosuppression and activating immune cells that can actively fight tumors (11, 24, 25). However, in addition to their expression in leukocytes, TLRs are found in multiple tumor types, including in OC, where their activation can have tumor-promoting effects (26–29). In fact, high levels of different TLRs in cancer cells have been associated with disease aggressiveness, treatment resistance, and poor clinical outcome. Most likely, this is a result of cytokine and chemokine-induced (e.g., as a result of NF-κB activation) recruitment of immunosuppressive and pro-angiogenic leukocytes to the tumor site (13, 30–32). In this mini review, we summarize recent studies and clinical trials aimed at exploiting TLR signaling pathways for OC immunotherapy.

## Toll-Like Receptor Signaling in Leukocytes

Toll-like receptors expressed in leukocytes (e.g., macrophages) serve a crucial function at the start of the immune response, activating numerous pro-inflammatory pathways resulting in cytokine secretion, and further activation of immune cells, including the adaptive immune response (21–23). It is known that the white blood cell population infiltrating the tumor environment differs between cancers and it has been established that the specific leukocyte profile at the site has a profound effect on tumor progression or regression (8, 30, 33). As the microenvironment of OC is typically immunosuppressive, efforts are ongoing to stimulate the immune population to effectively recognize and clear the tumor cells (12). In this regard, TLR activation in immune cells can favor the anti-tumor immune response, by increasing the capability of professional antigen-presenting cells (APCs) and facilitating the activation of anti-tumoral T cells (natural killer, NK cells; cytotoxic T lymphocytes, CTLs). In fact, the last decade of cancer immunology research has brought about several examples of the benefits of TLR activation in the immune cells surrounding the ovarian tumor milieu. Several clinical trials have been performed in an attempt to stimulate TLRs for OC therapy, including using TLR agonists in combination with other immunostimulating agents, such as DC vaccines (34, 35). Overall, these studies point to the potentially promising effects of TLR stimulation for OC patients with few efficacious treatment options available, especially if integrated with mainstream treatments or as adjuvants to other immunotherapies on a case-by-case basis.

In 2005, Adams et al. first described the rationale for TLR3 agonist therapy for advanced OC (35). In 2009, it was reported that TLR3 activation in DCs enhanced antigen processing and presentation by the APCs (24). Specifically, the authors described the inability of tumor-localized DCs to successfully activate anti-tumor immunity. Instead, they suppressed T cell function, although they were shown to be capable of processing tumor antigens. However, after stimulation with dsRNA (TLR3 ligand), with co-stimulation of CD40, DC function improved to trigger the desired tumor-eliminating inflammatory response. In these studies, this was indicated by the increase of interleukin 12 (IL-12) and type I IFN secretion by the DCs, as well as higher levels of co-stimulatory molecule expression and enhanced antigen-processing capability in both mouse and human OC samples. Furthermore, the treatment augmented the migratory capabilities of the DCs (to lymph nodes) and increased their antigen-presentation capability. These results point to the promising potential to re-structure the immunosuppressive OC environment to facilitate a robust anti-tumor response.

Earlier this year, Bellora and colleagues demonstrated that TLR activation in tumor-associated macrophages (TAMs) obtained from OC patients resulted in a shift from an M2 to an M1-polarization phenotype (36). This is significant, as M2-activated macrophages in the tumor environment are implicated in cancer growth, whereas M1-type (classically activated) macrophages are associated with better clinical outcome (37). M1-polarization is primarily immunostimulatory, characterized by the secretion of IL-12 and production of cytotoxic factors, such as nitric oxide (NO). M2-type or alternative macrophage activation largely results in immunosuppressive functions, and can be differentiated from M1-type activation by high levels of interleukin 10 (IL-10) secretion, as well as expression of specific markers, such as the mannose receptor (MR). In fact, the authors demonstrated that upon M1-polarization, the macrophages were able to induce cytolytic activity of NK cells (36). Thus, TLR activation in TAMs may be of clinical benefit by shifting the M2-polarized, immunosuppressive macrophages to a more immunostimulatory, anti-tumor phenotype.

Recently, a TLR8-specific agonist, VTX-2337 (Venti-RX Pharmaceuticals), entered Phase II clinical trials for OC patients with chemotherapy-resistant and recurring disease (38). The Phase I clinical trial with this agent was conducted in 2011 and was shown to be well-tolerated while exhibiting a dose-dependent therapeutic activity (39). The rationale for the therapy is to activate TLR8 in immune cells, whereby its signaling has been shown to have a suppressive effect on Tregs (40). Although the mechanism for the TLR8-dependent inhibition of this immune cell population is unclear, it is known to occur independently of DCs (41). In addition, TLR8 signaling appears to affect the morphology of NK cells, increasing their IFN-γ secretion, thereby strengthening the innate immune response (42). Furthermore, there have been implications for the potential of TLR7 stimulation for OC treatment (41, 43). In 2010, Geller and colleagues were the first to administer a selective small-molecule TLR7 agonist, 852A, to a small group of breast, ovarian, and cervical cancer patients with recurrent disease (43). Although significant side effects were observed with ~30% of those enrolled in the study discontinuing the therapy prior to completion, the authors showed immune activation and stabilization of disease in 2 of the 15 patients.

TLR9 ligands have similarly received interest as potential treatments for OC, specifically in combination with other immunomodulatory agents (44). In 2009, it was reported that a combinational treatment of CpG oligodeoxynucleotides (CpGODN), TLR9 ligand, and LL-37 (cathelicidin peptide) resulted in a better therapeutic outcome in mice. The authors demonstrated that the dual treatment increased the uptake of the TLR9 agonist CpODN (as TLR9 is endosomal). It was shown that the treatment increased the expansion and activation of NK cells in the murine peritoneal space, indicating an activation of innate immunity. Furthermore, studies assessing the potential role of the NK cells in the tumor environment revealed that they were heavily implicated in the observed anti-cancer effects of the therapy.

## Toll-Like Receptor Signaling in Ovarian Cancer Cells

In 2009, Zhou and colleagues reported on the expression of TLRs in human ovarian tissue samples, including both normal and neoplastic (benign and malignant) tissue (26). It was concluded that TLR2, TLR3, TLR4, and TLR5 were found on the epithelium of healthy ovary tissue. Additionally, this subset of TLRs was also expressed in a variety of human epithelial tumors and in numerous OC cell lines. The authors also found differential expression of TLR6 and TLR8 on all the samples, as well as low levels of TLR1, TLR7, and TLR9. It was demonstrated that the TLRs expressed in the epithelial cells were functional and it was suggested that their activation may constitute a mechanism by which the cancerous epithelial cells can manipulate inflammatory pathways to encourage tumor growth. The last decade of research on TLRs in tumor cells indicates that TLR activation in cancer cells generally results in increased production of cell survival and angiogenic molecules, as well as up-regulation of T-cell-suppressive factors, facilitating immune evasion. TLR signaling in ovarian has been attributed with more aggressive disease, potential for metastasis, and poorer end results in the clinic. Thus, specific inhibitors of TLRs (delivered to tumor cells) may be explored as potential therapeutic targets for some patients, especially in late-stage disease with fewer therapeutic options available (18). Recent research highlights the detrimental effects of TLR engagement in OC cells, indicating that inhibition of this receptor may be of benefit to the patient if targeted specifically in the cancer cells that overexpress the molecule.

The effects of TLR signaling in cancer cells have been extensively investigated for TLR4, perhaps the best-studied PRR. In 2005, Huang and colleagues reported on its expression and activation in numerous mouse cancer cell lines (45). They determined that TLR4 stimulation by LPS in tumor cells increased production of numerous soluble factors, such as IL-6, and ultimately inhibited the ability of CTLs to recognize and kill the cancer cells. It was also found that LPS treatment of the murine tumor cell supernatants impeded the proliferation of T cells and inhibited NK cell activity. Further, the authors demonstrated that inhibition of TLR4 signaling in tumor cells significantly increased survival in animal studies. The menacing effects of TLR4 activation specifically on human OC progression have also been reported (46). Kelly et al. demonstrated that TLR4 is upregulated in numerous ovarian epithelial tumors and that high expression correlates with increased tumor progression and likelihood of developing chemo-resistance to Paclitaxel. Additionally, TLR4 (and subsequent NF-κB) activation has been demonstrated for human ovarian granulosa tumor cells (47). Thus, TLR4 inhibition in several types of OC cells may be therapeutically beneficial in conjunction with standard chemotherapy in an effort to decrease the likelihood of drug resistance.

Similarly, TLR9 signaling by OC cells (as well as breast cancer cells) has been associated with disease aggressiveness and poor clinical outcome (48). Berger and colleagues determined that higher levels of TLR9 expression correlated with more severe tumor grade. Consistently, *in vitro* scratch essays revealed the increased migratory capabilities of tumor cells expressing higher TLR9 levels (in both ovarian and breast tumor cells). It was also reported that higher TLR9 expression was more common in poorly differentiated tumors (hormone-receptor-negative tumor cells were found to have more TLR9); thus, these tumors have fewer targeted therapeutic options. In addition, it was found that OC patients with metastatic disease had elevated levels of hypo-methylated DNA (TLR9 ligand) in their serum. Further, the authors offered even more evidence of the detrimental effects of TLR9 signaling in OC cells, showing the co-localization of TLR9 and its ligand, as well as NF-κB activation, which was proportional to the levels of TLR9 expression. Significantly, NF-κB appears to be constitutively activated in numerous cancer types, whereby it is associated with highly aggressive disease and poor disease outcome, highlighting the potential of TLR targeting to inhibit this important inflammatory switch in tumor cells (28, 49).

## Endogenous TLR Ligands and Implications for Cancer Therapy

In addition to the PAMPs that can activate TLRs (e.g., LPS, viral RNA, etc.), endogenous ligands for these molecules have also been identified (50). For instance, TLR2 and TLR4 can be triggered by biglycan and endoplasmin, while nucleic acid-sensing TLRs can bind to mRNA (TLR3), as well as siRNA (TLR7, TLR8). Additionally, damage-associated molecular patterns (DAMPs), molecules induced during cell stress or damage (e.g., HMGB1) can activate TLRs (51–53). As discussed, attempts to harness TLRs to promote cancer regression have been attempted in numerous trials, where the treatments are often used in combination with standard chemotherapy or radiation practices in an effort to maximize patient response. In fact, it appears likely that cell death (e.g., necrosis from standard therapy) can result in release of endogenous TLR ligands, which may activate nearby leukocytes, potentially improving the anti-tumor response (50). Continued characterization of ligands and determining downstream signaling will help elucidate the full function of TLRs in cancer progression and give more direction for novel therapeutic strategies for specific cancer types.

## Concluding Remarks

**Figure 1:**
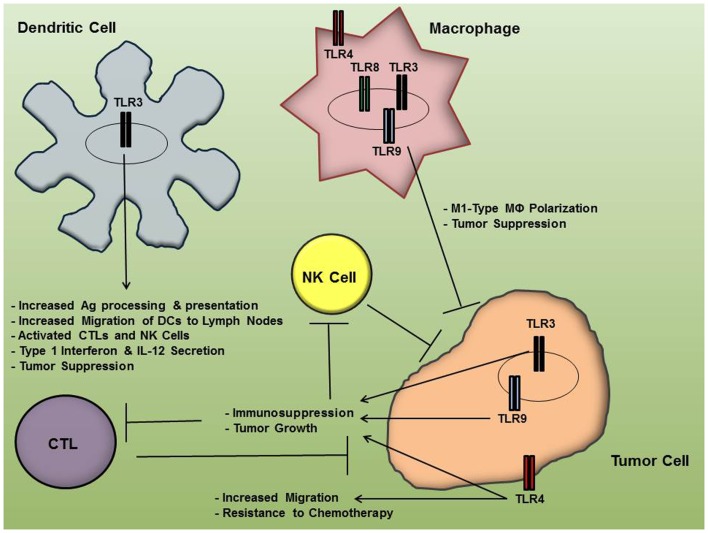
**Toll-like receptor (TLR) activation in ovarian cancer cells and immune cells results in differential effects on tumor progression**. While TLR engagement in immune cells may facilitate an anti-tumor inflammatory microenvironment, their signaling pathways in tumor cells may result in immunosuppression and resistance to chemotherapy, thereby furthering tumor growth.

The last decade of research on TLR activity and its implications in OC progression indicate that inhibition of certain TLRs in cancer cells and/or TLR stimulation in immune cells may be of therapeutic benefit in some patients. While immune activation by means of TLR stimulation can generate an anti-cancer effect, the cytokine profile following TLR activation in tumor cells typically favors an immunosuppression that can potentiate immune-tolerance and promote angiogenesis, furthering tumor growth. Figure 1 summarizes the differential effects of TLR signaling by OC cells and immune cells. Undoubtedly, TLR targeting is a promising area of research for OC and other malignancies, although these pathways can produce such varying effects that exploitation of TLR pathways for cancer therapy has frequently been referred to as a “double-edged sword” (54, 55). Therefore, TLR targeting for OC therapy must be pursued with care and stimulating or inhibiting agents be delivered in a cell-specific manner. Given the complex nature of the effects of TLR activation in various cells, much remains to be investigated, including the multiple regulators of TLR expression and activation in the different cell types. For instance, miRNAs have recently been shown to be “fine-tuning” regulators of TLR signaling pathways; thus further research in this exciting area of study may yield even more targeting opportunities for TLR regulation that could be applied in cancer therapy (56, 57). Finally, future therapeutic strategies may be realized more effectively in conjunction with novel drug delivery mechanisms that allow for more cell-specific drug targeting.

## Conflict of Interest Statement

The authors declare that the research was conducted in the absence of any commercial or financial relationships that could be construed as a potential conflict of interest.
